# Progression of ‘OMICS’ methodologies for understanding the pathogenicity of *Corynebacterium pseudotuberculosis*: the Brazilian experience

**DOI:** 10.5936/csbj.201303013

**Published:** 2013-10-13

**Authors:** Fernanda A. Dorella, Alfonso Gala-Garcia, Anne C. Pinto, Boutros Sarrouh, Camila A. Antunes, Dayana Ribeiro, Flavia F. Aburjaile, Karina K. Fiaux, Luis C. Guimarães, Núbia Seyffert, Rachid A. El-Aouar, Renata Silva, Syed S. Hassan, Thiago L. P. Castro, Wanderson S. Marques, Rommel Ramos, Adriana Carneiro, Pablo de Sá, Anderson Miyoshi, Vasco Azevedo, Artur Silva

**Affiliations:** a Laboratório de Genética Celular e Molecular, Instituto de Ciências Biológicas, Universidade Federal de Minas Gerais, Brazil; b Instituto de Ciências Biológicas, Universidade Federal do Pará, Belém-PA, Brazil

**Keywords:** *Corynebacterium pseudotuberculosis*, SOLiD next generation sequencing, Ion Torrent next generation sequencing, SDS-PAGE, mass spectrometry, RNA-seq

## Abstract

Since the first successful attempt at sequencing the *Corynebacterium pseudotuberculosis* genome, large amounts of genomic, transcriptomic and proteomic data have been generated. *C. pseudotuberculosis* is an interesting bacterium due to its great zoonotic potential and because it causes considerable economic losses worldwide. Furthermore, different strains of *C. pseudotuberculosis* are capable of causing various diseases in different hosts. Currently, we seek information about the phylogenetic relationships between different strains of *C. pseudotuberculosis* isolates from different hosts across the world and to employ these data to develop tools to diagnose and eradicate the diseases these strains cause. In this review, we present the latest findings on *C. pseudotuberculosis* that have been obtained with the most advanced techniques for sequencing and genomic organization. We also discuss the development of *in silico* tools for processing these data to prompt a better understanding of this pathogen.

## Background

*Corynebacterium pseudotuberculosis* is a Gram-positive facultative intracellular pathogen that belongs to the class *Actinobacteria*. This pathogen is aerobic, has mycolic acid in its cell wall, displays pleomorphic forms, does not sporulate or encapsulate, is non-motile and possesses fimbriae. *C. pseudotuberculosis* has great infectious potential and implications for zoonotic transmission, as it affects goats, sheep, horses, buffaloes, camels, cattle and primates, causing different symptoms. Furthermore, it has already been reported as the causative agent of more than 33 cases of infection in humans. *C. pseudotuberculosis* presents two biovars, ovis (nitrate negative reduction) and equi (nitrate positive reduction); the former biovar is mainly associated with the globally-distributed disease Caseous Lymph Adenitis (CLA), which affects the lymph nodes and visceral organs of goats and sheep and causes economic losses by compromising the skin, weight, milk and meat production of the animals as well as causing death and compromising the carcass. Although many vaccines exist, they are mainly intended for use in sheep and goats and provide variable levels of protection [1–3].

*C. pseudotuberculosis* has interesting survival mechanisms and is able to utilize different strategies to adapt to its environment. Once it is successfully established within a host and able to replicate inside phagocytic cells, this pathogen will evade the immune system with apparent ease. As a result, chronic infections may last for most, if not all, of an animal's life [3]. In the present review, we report the latest information regarding the genomic, proteomic and transcriptomic features of this interesting microorganism, and we attempt to correlate such information with its virulence and pathogenicity.

## Genomics

### The beginning of the *C. pseudotuberculosis* genome projects

The first attempt to identify the genomic sequence of *C. pseudotuberculosis* was performed by Dorella and collaborators [4], in which genomic libraries of the 1002 strain of this species were constructed using a bacterial artificial chromosome (BAC) vector. This high-quality genomic library, containing approximately 1,800 clones, harbored inserts ranging from 24.5-121 kbp. Partial characterization of this library through a BAC end-sequencing strategy, namely the identification of genome survey sequences (GSS), generated 215 GSS at relatively low cost; these were deposited on the NCBI website. Using these sequences for *in silico* analysis, it was possible to identify putative genes involved in virulence based on their similarity to other deposited sequences and generate a catalog of genes, such as the putative siderophore-binding protein (GSS number BH740428) that increased our biological knowledge of the microorganism. The high quality, low redundancy and absence of contaminants in the library, together with the large number of clones it contained, permitted this library to serve as a physical map for the characterization of the *C. pseudotuberculosis* genome. Moreover, library characterization also allowed for confirmation of the close phylogenetic relationship between *C. pseudotuberculosis* and *C. diphtheriae*, *C. glutamicum*, *C. efficiens* and *C. jeikeium* [4]. Based on this initiative, a project to sequence the first entire genome of *C. pseudotuberculosis* 1002 strain was started by the Rede Genoma de Minas Gerais (RGMG-Brazil) in 2006 and concluded in 2009. This genome was sequenced using the Sanger di-deoxy method and has now been assembled, annotated and deposited in the NCBI database under accession number CP001809.

### The challenge of next-generation sequencing

The *C. pseudotuberculosis* genome project has expanded its boundaries and today the network includes the Rede Paraense de Genômica e Proteômica, which has worked with all of the versions of SOLiD™ (Life Technologies) since v.2 and now employs the most advanced next-generation sequencing (NGS) platforms: the SOLiD™ 5500 series (Life Technologies) and Ion Torrent PGM (Life Technologies). These NGS platforms can sequence more than one bacterial genome per day, thus demonstrating the feasibility of sequencing *C. pseudotuberculosis* strains. This important partnership has also contributed computational resources to process the huge amount of data generated by these new DNA-sequencing technologies.

To date, fifteen strains of *C. pseudotuberculosis* have been sequenced (Table 1), employing all of the presently available technologies. Based on the data obtained by sequencing, the average G + C content of all 15 strains is 52.2%; each genome has an average of approximately 2,195 genes, and total genome sizes range from 2.28 to 2.34 Mb. The sequencing of several strains of *C. pseudotuberculosis* is paving the way for further studies. In 2011, Barh and colleagues compared four genomes of *C. pseudotuberculosis* (strains FRC41, 1002, C231 and I19) with eight other sequenced genomes of pathogens belonging to a group that includes genera such *Corynebacterium, Mycobacterium, Nocardia* and *Rhodococcus*, which are commonly found in humans, goats, sheep, cattle and horses [5]. As a result of this comparative genomic analysis, potential molecular targets were identified for the production of drugs and vaccines.

**Table 1:** Strains of *Corynebacterium pseudotuberculosis* that were deposited in NCBI between 2009 and 2012 and their structural characteristics.

Strain	Biovar	Animal/ Host	Site of Isolation	Country of Isolation	Sequencing Technology	Chromosome	Size (Mb)	GC%	Genes	Proteins
1002	*ovis*	Goat	Abscess	Brazil, Bahia	454, Sanger	1	2.34	52.2	2,203	2,090
C231	*ovis*	Sheep	Abscess	Australia	454	1	2.33	52.2	2,204	2,091
162	*equi*	Camel	Abscess	UK	SOLiD v3	1	2.29	52	2,150	2,002
258	*equi*	Horse	Not specified	Belgium	SOLiD v3	1	2.31	52.1	2,195	2,088
CIP5297	*equi*	Horse	Not specified	Kenya	SOLiD v2	1	2.32	52.1	2,194	2,060
PAT10	*ovis*	Sheep	Abscess	Patagonia	SOLiD v2	1	2.34	52.2	2,200	2,079
I19	*ovis*	Bovine	Not specified	Israel	SOLiD v2	1	2.34	52.2	2,213	2,095
31	*equi*	Buffalo	Not specified	Egypt	Ion Torrent, SOLiD v3	1	2.34	52.2	2,170	2,063
FRC41	*ovis*	Human	Inguinal lymph node	France	454	1	2.34	52.2	2,171	2,110
267	*ovis*	Llama	Submandibular abscess	California	SOLiD v3	1	2.34	52.2	2,249	2,148
316	*equi*	Horse	Subcutaneous abscess	California	Ion Torrent	1	2.31	52.1	2,234	2,106
01/06	*equi*	Horse	Abscess	California	Illumina	1	2.28	52.2	2,127	1,963
3/99-5	*ovis*	Sheep	Abscess	Scotland	Illumina	1	2.34	52.2	2,239	2,142
42/02	*ovis*	Sheep	Abscess	Australia	Illumina	1	2.34	52.2	2,164	2,051
P54B96	*ovis*	Antelope	Liver, lung, mediastinal lymph node	South Africa	Ion Torrent, SOLiD v3	1	2.34	52.2	2,205	2,084

The study of the diversity among strains promotes our understanding of gene rearrangement, genomic plasticity as loss and gains and inversions in the genome. In addition, this research provides valuable information regarding molecular epidemiology, microevolution, lineage-specific genes and common genes among the isolates [6], contributing to the development of new therapies that are more effective for the control of caseous lymphadenitis (CLA).

### Structural genome

Of the fifteen genomes deposited at NCBI, nine belong to the biovar ovis, and six belong to the biovar equi (Table 1). While the ovis strains have almost no genetic differences, the grouping of the equi strains appears to be asymmetric in relation to biovar ovis. Therefore, it is important to detect the differences between *ovis* and *equi* to develop a common vaccine or diagnostic tool for all of them. Typically, vaccines against *C. pseudotuberculosis* infection designed for sheep do not have equal efficacy in goats, although both species are usually infected by bacteria belonging to the biovar *ovis*. Thus, the vaccines developed for *C. pseudotuberculosis* biovar *ovis* may not have the same efficacy in hosts infected with biovar *equi*, which further complicates treatment of *C. pseudotuberculosis* by different animal breeders [1].

Interestingly, no major differences between the structural characteristics of biovars *equi* and *ovis* have been observed, such as the numbers of CDS, genes or proteins, which are very similar between strains of both biovars (Table 1). The differential pathogenicities of the biovars might be due to the presence of genes that are strain-specific, as each pathogen appears to preferentially infect particular hosts, therefore causing different disease symptoms. Thus, specific genes and other unknown process may underlie host preference and determine the different symptoms of the infection process [1].

Features that are common among all of the strains are GC content and the number of ribosomal clusters. GC content is related to different intrinsic or extrinsic factors, and a high GC content suggests that the genetic material has greater stability, providing a more robust genome that suffers less from the influence of environmental variations [7].

With regard to the number of rDNA operons, all strains present four copies, and each ribosome consists of one 5S, one 16S and one 23S. This fact may possibly be related to the slower replication of *C. pseudotuberculosis* compared to *Escherichia coli*, which has seven copies of the rDNA operon, or *C. glutamicum*, which has six copies, considering that ribosomal operons can perform diverse functions related to the control of protein synthesis [8].

### Software and databases for Corynebacterium pseudotuberculosis genome analysis

A rapid increase in the number of complete genomes over the past few decades in the form of large molecular datasets in public databases has provoked researchers to develop numerous computational tools and public or proprietary databases. These holistic approaches have facilitated the rapid study and understanding of the innumerable biological functions that are encoded by genomic DNA. The barrier to unraveling prokaryotic genomes has been eliminated using the next generation of high-throughput sequencing technologies, such as SOLiD, GS FLX, Ion Torrent PGM and Illumina, which have prominent advantages over Sanger sequencing. However, although these technologies significantly reduce the cost and time for genome sequencing, they still pose challenges for various aspects of data processing and analysis, such as the assembly of short reads [9]. A number of user-friendly interfaces and stand-alone computational tools have been developed to evaluate the genomic and transcriptomic data obtained from these high-throughput platforms.

Presently, bioinformaticians have developed and are further revising some useful tools and software packages using different algorithms and in-house scripts. A brief description and application of each software program for the data analysis of *C. pseudotuberculosis* and/or taxonomically related organisms is presented below.

1- Pathogenicity Island-Prediction Software (PIPS):

This software is designed to predict the pathogenicity islands (PAIs) in bacterial genomes, utilizing multiple features in an integrative manner. PAIs are large genomic regions acquired through horizontal gene transfer, which have in common the following: deviations in G + C content and codon usage, the presence of transposase and virulence factors, flanking insertion sequences and/or tRNA genes and their absence in non-pathogenic organisms of the same genus or related species. PIPS uses these multiple features to detect PAIs. For validation purposes, PIPS was utilized with model organisms of the genera *Corynebacterium* and *Escherichia*, and the results showed that PIPS provided better accuracy (85-88%) and superior efficiency compared with the other available software tools. This software is easy to install on a personal computer and provides a user-friendly interface for students and researchers [10].

2- Quality Assessment Software (QA):

This software is used to analyze the quality of sequence reads from next-generation platforms. The software removes the reads, which present average quality below the Phred quality cutoffs. The process of quality filtering reduces miss-assemblies and incorrect mapping against the reference genome that are attributable to low quality sequences from the raw data. The software helps to review graphs that show the distribution of quality values from the sequencing reads, including the average and the accumulated quality for each base. Libraries of fragments from SOLiD sequencing of *C. pseudotuberculosis* (Cp162) and *Exiguobacterium antarcticum* (B7) were used as sample data to test the software. QA is a Java-based program that is available at http://qualevaluato.sourceforge.net [11]. A new version of this software, called Quality Assessment Long Reads [12], was developed to apply the Phred quality filter over Ion Torrent PGM data due to the read length: ≈120 bp for the first release of the platform and ≈400 bp with a recent protocol.

3- Singular Value Decomposition (SVD):

This is a very useful technique for information retrieval that helps to uncover the relationships between elements that are not prima facie related. In turn, this leads to the improved inference of evolutionary relationships between amino acid sequences of different species. SVD produces a revised distance matrix for a set of related elements and provides results resembling the internationally accepted scientific gold standard of Linnaean taxonomy. The SVD-based computations establish non-obvious, relevant relationships among the clustered elements, providing a deterministic method for grouping related species. This approach was initially developed to reduce the time needed for information retrieval and analysis of very large-scale genome and proteome data sets in the complex Internet environment. The results obtained by this technique are in close approximation with results based on Linnaean taxonomy, which indicates that SVD can indicate evolutionary relationships of species and construct better quality clusters and phylogenetic trees [13].

The analysis of prokaryotic genomes can be further aided with new algorithmic methods and tools and advancements in bioinformatics and computational biology. These techniques will provide more opportunities to study in detail the “OMICS” of specific organisms. Unifying current and upcoming computational resources to provide a global and integral picture of biology is important and can be achieved by mutual cooperation among researchers from distinct areas.

4- Core StImulon (CSI)

One methodology for performing RNA sequencing (RNA-seq) analyses is the *de novo* approach, which is commonly used when reference genomes are not available in biological databases. An important feature of this method is that it identifies shared transcripts among stimulons (i.e., the set of expressed genes under a given condition), which can permit the selection of possible candidates for vaccine studies through searches for the specific genes of an organism in addition to permitting the identification of new transcripts that have not been previously annotated. We sequenced the cDNA of *Corynebacterium pseudotuberculosis* strain 1002 using the SOLiD V3 system under the following conditions: osmotic stress (2 M), acidity (low pH), heat shock (50°C) and a control condition. To identify the transcripts that were shared among the stimulons and integrate this information with the BLAST and BLAST2GO results, the software CoreStImulon (CSI) was developed, which allows genes to be characterized in terms of their ontology [14].

5- FunSys

FunSys software, which is a stand-alone tool with a user-friendly interface, was developed to evaluate and correlate the differential expression profiles from RNA-seq and proteomics datasets. FunSys produces charts and reports based on the results of the analysis of differential expression (generated using other software) to aid in the interpretation of the results [15].

## Proteomics

### Proteomics of C. pseudotuberculosis

Unlike genomic studies, proteomics evaluates the protein profile of a cell, tissue or organism [16, 17]. Proteomic studies can provide valuable information about changes in protein synthesis, post-translational modifications and protein-protein interactions, thereby increasing knowledge of physiological phenomena for a specific condition and helping to establish a fundamental understanding of an organism's cellular physiology and virulence factors [16, 18]. The global expression of bacterial proteins is required for growth, survival or pathogenicity, and cataloguing these proteins in response to a determined condition is a key step toward understanding the physiology of these microorganisms [18, 19]. To identify virulence factors and obtain further information about the biology of pathogenic bacteria, studies have been performed using proteomics to characterize whole cells, cytoplasmic and membrane proteomes and the secretome/exoproteome of these pathogens [18].

The primary proteomic studies involving *C. pseudotuberculosis* were intended to analyze the extracellular protein fraction. This protein fraction is associated with the uptake of nutrients, cell-to-cell communication, proteolysis, hemolysis, detoxification, escape from the immune system and destruction of competing microorganisms in their respective environments. However, during the process of adaptation and survival in hostile environments, pathogenic bacteria need to secrete different molecules for adhesion, invasion, proliferation and survival in the host cell [20, 21]. Thus, the study of extracellular proteins is a useful strategy to identify new virulence factors and target immunogenics [22].

Initially, with the aim of identifying new targets for the development of immunodiagnostics and vaccine targets to combat CLA, various research groups conducted proteomic studies using one-dimensional electrophoresis based on sodium dodecyl sulfate polyacrylamide gel electrophoresis (SDS-PAGE) and immunoblotting to characterize the whole cell fraction and extracellular proteins of *C. pseudotuberculosis*. The bacteria in these studies were grown in complex media containing exogenous proteins that would contaminate extractions of extracellular proteins [23, 24]. Studies showed that the use of chemically defined medium (CDM) is an effective strategy to identify bacterial components for therapeutic applications [25]. In this context, a CDM for *C. pseudotuberculosis* growth in macromolecule-free conditions was developed [26]. The evaluation of humoral and cellular immune responses of goats experimentally infected with *C. pseudotuberculosis* showed that interferon-γ (IFN- γ) detection using excreted-secreted antigen after cultivation of this pathogen in CDM provided more specific results compared with the use of whole cell sonicated antigen [27]. This suggested that the bacterial growth in CMD and use of the secreted protein fraction may be an interesting strategy for the study of immunogenic proteins of *C. pseudotuberculosis*.

To optimize the process of obtaining the extracellular fraction, Paule and colleagues [27] established an efficient protocol for extracting the extracellular proteins of *C. pseudotuberculosis* based on the three-phase partitioning (TPP) technique. After analyzing the protein extract by SDS-PAGE and immunoblotting, it was possible to detect proteins that were not detected in previous studies [28]. Notably, all of the results obtained by Paule and colleagues [28] only indicated the molecular weights of the proteins or reactivity of the proteins against the sera of infected animals without protein characterization by mass spectrometry (MS).

The *C. pseudotuberculosis* genome project [29] generated information about the pathogenicity and virulence of this microorganism. From the genomic data, the *in silico* pan-exoproteome of *C. pseudotuberculosis* has been deduced [30]. However, how these gene products interact and what their functions are in physiological processes must be elucidated. To respond to these questions and validate gene annotations, proteomic approaches have been applied to characterize the exoproteome of *C. pseudotuberculosis*.

### Comparative proteomics

Studies have demonstrated that comparative proteomics is a powerful strategy to characterize bacterial proteomes, and thus it has been adopted to characterize the proteomes of various pathogenic bacteria [21, 31, 32]. A comparative proteomic study was conducted using the “shotgun proteomics” approach to characterize the exoproteome of two strains, *Cp1002* and *CpC231*, of *C. pseudotuberculosis*, both of which belong to the biovar *ovis* but were isolated from different hosts (goat and sheep, respectively). This study combined the techniques of TPP [27] and gel-free separation using liquid chromatography coupled with mass spectrometry (LC-MS), called TPP-LC/MS^E^ [33]. The two strains were maintained on BHI agar or in broth and in CDM to study proteome growth. The results obtained from this work showed quantitative and qualitative changes between the exoproteomes of both strains. Furthermore, this strategy permitted the characterization of 93 extracellular proteins of *C. pseudotuberculosis* that were associated with the physiology and virulence of this pathogen [33]. The identified proteins that play a role in virulence include phospholipase D (PLD), the main virulence factor of *C. pseudotuberculosis*, which is associated with the spread of bacteria within the host [34]; iron siderophore binding protein (FagD), a component of an iron uptake system [35]; and serine proteinase (CP40), which showed protective activity against infection by *C. pseudotuberculosis* [36]. However, these proteins were identified only in the extracellular proteome of *CpC231*, suggesting that these proteins may not be secreted by *Cp1002*, which may influence the pathogenesis of this strain [33].

Another approach that has been employed to analyze the exoproteome of *C. pseudotuberculosis* is serological proteome analysis (SERPA), which involves 2-DE immunoblotting and identification of antigenic spots by an MS technique. This strategy has been applied to several pathogenic bacterial species to identify virulence factors, target the development of drugs and vaccines and conduct immunodiagnostics [37, 38]. In this context, Seyffert et al. [39] conducted a preliminary serological secretome analysis of *C. pseudotuberculosis* and evaluated the exoproteome of strain 1002 *ovis*. The use of the SERPA approach enabled the characterization of six immunoreactive proteins against the serum of animals infected with *C. pseudotuberculosis*. These identified proteins represent potential targets for developing vaccine targets and diagnostics to combat CLA.

Currently, with advances in proteomic studies, new techniques have been developed and applied for the study of several pathogens. Thus, the application of different proteomic approaches is a powerful strategy to characterize the proteome of *C. pseudotuberculosis* and broaden our knowledge of the physiology and pathogenesis of this pathogen.

## Transcriptomics

The mechanisms with which pathogenic microorganisms surpass the hostile conditions found in a host are of great importance for successful infection, and the genes related to such adaptations constitute clear targets for the development of new diagnostics and vaccines. The advent of RNA microarrays and high-throughput RNA-seq technologies has allowed not only the comprehensive assessment of differential gene expression in bacteria but also the identification of genetic structures such as operons, transcriptional start sites, non-coding regulatory RNAs and small RNAs [40].

Similar to *M. tuberculosis*, *C. pseudotuberculosis* infects and persists inside macrophages, although it does not prevent fusion between the phagosome and lysosome. Because this bacterium is subjected to different stresses in the phagolysosome, Pinto and colleagues [14] evaluated its transcriptome following *in vitro* exposure to high osmolarity (sodium chloride at a final concentration of 2 M), heat shock (50°C) or acidic pH (5.0) by performing RNA-seq with SOLiD technology. When the sets of genes expressed only under each stress condition, which together compose the core stimulon of *C. pseudotuberculosis*, were examined, most of the targets identified were related to oxidation and reduction events, while cell division and the cell cycle were the second- and third-most upregulated processes, respectively. According to the Gene Ontology database, some of the genes in the core stimulon are directly involved in stress responses; one example involves an encoder of a two-component system response-regulator protein that is also linked to pathogenesis. Other genes highlighted by the authors include *dps* (a gene involved in resistance to oxidative stress) and a gene that encodes for one component of the ABC-type iron-uptake system.

The assessment of global transcriptional profiles in bacteria constitutes a key strategy for unveiling mechanisms that are important for virulence and pathogenicity; thus, any efforts to increase the feasibility of RNA-seq experiments are welcome when studying pathogens such as *C. pseudotuberculosis*. Because large portions of sequencing reads are mapped to ribosomal RNA genes, Castro and colleagues [41] tested a new methodology, based on denaturing high-performance liquid chromatography, to deplete ribosomal transcripts from bacterial total RNA samples using *C. pseudotuberculosis* (biovar *equi*) as a model organism. With the elimination of 78% to 92% of rRNA, which are levels that resemble those obtained with a conventional subtraction kit, this new method offers financial advantages for researchers who have access to a chromatographic system.

The elucidation of which gene products of *C. pseudotuberculosis* are directly involved in survival and adaptation during infection has yet to come. As global gene expression profiling will most likely provide key knowledge for the development of effective prophylactic measures in the future, researchers will certainly take a step ahead by integrating information from both transcriptomic and proteomic approaches.

## Future of this field

Studies of prokaryotic genomes, transcriptomes and proteomes have been considerably improved with the development of new experimental methods, algorithms and tools and advances in bioinformatics and computational biology.

The main objective of these studies is to find clues that may be useful in developing a vaccine and a diagnostic approach that is effective for all hosts that suffer from *C. pseudotuberculosis* infection. Another goal is to elucidate the physiology, pathogenicity and virulence mechanisms of this bacterium.

In response to advances in molecular biology in the last few years, much information regarding biological systems has been elucidated via a variety of genome-sequencing projects. However, sequencing reveals little about how the proteins of an organism operate individually or together to perform their functions. The integration of both current and upcoming resources to provide a global and integral biological picture is important and can be achieved by mutual cooperation between researchers from distinct areas.
